# The phenotype of recovery XI: associations of sleep quality and perceived stress with discounting and quality of life in substance use recovery

**DOI:** 10.1007/s11136-024-03625-z

**Published:** 2024-03-20

**Authors:** Yu-Hua Yeh, Michelle H. Zheng, Allison N. Tegge, Liqa N. Athamneh, Roberta Freitas-Lemos, Candice L. Dwyer, Warren K. Bickel

**Affiliations:** 1 Illinois College, Jacksonville, IL USA; 2 Civil and Environmental Engineering Department, University of California, Los Angeles, CA USA; 3 Fralin Biomedical Research Institute at VTC, Roanoke, VA USA; 4 Department of Statistics, Virginia Tech, Blacksburg, VA USA; 5 Department of Psychology, Virginia Tech, Blacksburg, VA USA

**Keywords:** Sleep, Stress, Delay discounting, Quality of life, Addiction recovery

## Abstract

**Purpose:**

Sleep and stress show an interdependent relationship in physiology, and both are known risk factors for relapse in substance use disorder (SUD) recovery. However, sleep and stress are often investigated independently in addiction research. In this exploratory study, the associations of sleep quality and perceived stress with delay discounting (DD), effort discounting (ED), and quality of life (QOL) were examined concomitantly to determine their role in addiction recovery. DD has been proposed as a prognostic indicator of SUD treatment response, ED is hypothesized to be relevant to the effort to overcome addiction, and QOL is an important component in addiction recovery.

**Method:**

An online sample of 118 individuals recovering from SUDs was collected through the International Quit and Recovery Registry. Exhaustive model selection, using the Bayesian Information Criterion to determine the optimal multiple linear model, was conducted to identify variables (i.e., sleep quality, perceived stress, and demographics) contributing to the total variance in DD, ED, and QOL.

**Results:**

After model selection, sleep was found to be significantly associated with DD. Stress was found to be significantly associated with psychological health, social relationships, and environment QOL. Both sleep and stress were found to be significantly associated with physical health QOL. Neither sleep nor stress was supported as an explanatory variable of ED.

**Conclusion:**

Together, these findings suggest sleep and stress contribute uniquely to the process of addiction recovery. Considering both factors when designing interventions and planning for future research is recommended.

## Plain English summary

In this exploratory study, we investigated the relationship between sleep, stress, and addiction recovery. Sleep and stress are known to make it harder to recover from substance use disorders, but researchers often study them separately. We recruited participants from the International Quit & Recovery Registry and asked them to complete an online survey about their sleep quality and perceived stress, along with variables relevant to addiction recovery. We found that sleep was related to how much people valued rewards based on the associated delay, while stress was related to quality of life regarding psychological health, social relationships, and environment. Both sleep and stress were related to physical health. However, we did not find any evidence to suggest that either sleep or stress was related to how much people valued rewards based on the associated effort. These findings highlight the importance of considering both sleep and stress in designing effective interventions for addiction.

## Introduction

Substance use disorders (SUDs) are a worldwide public health concern. Although evidence-based treatments for SUDs are effective compared to placebo, studies have shown that about 40 to 60 percent of those who received treatments relapsed [1–3]. Given that recovery from SUDs often involves preventing and/or managing relapses, identifying factors that may facilitate this process is imperative. Among these, sleep and stress are particularly pertinent, having been recognized as relevant factors for SUD relapse [4, 5] and treatment targets in recovery [6, 7]. However, their relationships to other known factors in addiction recovery, such as delay discounting (DD), effort discounting (ED), and quality of life (QOL), are less well understood [8–11]. As part of a broader effort to phenotype recovery, this exploratory study examined the impact of sleep quality and perceived stress on DD, ED, and QOL among individuals in recovery from SUDs.

Sleep and stress have been extensively investigated over the course of SUDs. Sleep disturbance can be a result of drug abuse and is experienced as a withdrawal symptom during the quitting period [12–14]. Stress can increase the susceptibility to drug abuse and increase the risk of developing SUDs [15–17]. Both sleep and stress are associated with psychological distress among individuals with SUDs, are risk factors for relapse, and have been targeted in SUD treatments [4–7, 13, 18–23]. Noticeably, sleep and stress show an interdependent relationship in physiology [24] and have been investigated with substance use in research on other psychopathologies (e.g., post-traumatic stress disorder) [25]. However, they have yet to be examined together among individuals in recovery from SUDs. As a result, whether they each explain unique variance in SUD recovery determinants such as DD and QOL remains unknown to date.

Delay discounting, the decline in the present value of a reward with delay to its receipt captures important human decision-making processes and is robustly associated with SUDs [26–29]. Recent studies suggested that characterization of DD is a prognostic indicator of treatment response, as high DD rates were predictive of treatment retention and post-treatment relapse and abstinence [11, 30–33]. Due to its relevance to health, the associations of sleep and stress with DD have been investigated, although the findings regarding sleep are mixed to date. In most studies that involved short-term sleep deprivation in healthy adults, the correlation between sleep and DD rates was nonsignificant [34–38]. A recent study involving a large sample size of healthy young adults (*n* = 1,190) found a positive correlation between self-reported habitual, long-term sleep deprivation and monetary DD rates [39]. However, another study with college students (n = 297) did not show a similar correlation [40].

In contrast to the mixed findings on sleep, a positive correlation between stress and DD rates has been established and observed in the addiction recovery population [41–44]. Moreover, imagining stressful events significantly increased DD rates in individuals with alcohol use disorder [45], suggesting a direct causal relationship. Noticeably, the abovementioned findings on the association between DD and sleep were from investigations of other populations. To our knowledge, no study in addiction recovery has concomitantly considered both sleep and stress.

The current study also examined the role of sleep and stress in ED, which measures the devaluation of rewards with physical effort [46]. Although research on ED is limited, studies found that alcohol use disorder severity is positively correlated with the willingness to exert effort to obtain alcohol, and short-term nicotine deprivation is associated with greater effort to secure cigarette alternatives [47, 48]. The relevance of ED to addiction recovery has been hypothesized, noting overcoming addiction, remaining abstinent, and pursuing non-drug activities is effortful [9]. Furthermore, studies in healthy adults and animals have shown that sleep deprivation, fatigue, and acute stressors increased ED rates [36, 49–52].

In addition to reductions in use, one goal of addiction recovery is to improve QOL [53, 54]. The definition of QOL provided by the World Health Organization (WHO) is “an individual’s perception of their position in life in the context of the culture and value systems in which they live and in relation to their goals, expectations, standards, and concerns” [55]. The construct of QOL incorporates the individual’s subjective view of clinical, functional, and personal variables and hence, is relevant to addiction recovery [8, 56]. Both sleep and stress have been shown to impact QOL [57–62]. Moreover, poor sleep quality was linked to lower QOL in individuals with SUDs [63, 64]. However, to our knowledge, no study has concomitantly examined the impact of sleep and stress on QOL in individuals recovering from SUDs.

This study aimed to examine the associations of sleep quality and perceived stress with DD, ED, and QOL among individuals in recovery from SUDs. Based on previous research, we hypothesized that sleep quality and perceived stress would be significantly associated with DD, ED, and QOL in univariate regression analyses. To further explore these associations and determine whether sleep quality and perceived stress each play a unique role in the variables of interest, we conducted exhaustive model selections to identify variables (i.e., sleep quality, perceived stress, and demographics) contributing to the total variance in DD, ED, and QOL in the optimal multiple linear model. Understanding the intricate interplay between sleep quality, perceived stress, and these specific domains is vital, given the recognized importance of addressing sleep and stress in SUD treatment. This knowledge can illuminate the underlying mechanisms at play and pave the way for the development of personalized interventions.

## Methods

### Participants

Participants were recruited from the International Quit and Recovery Registry (IQRR; quitandrecovery.org), an online addiction recovery community for individuals who are 18 years or older and self-identify as being in recovery from substance misuse. The IQRR distributed quarterly assessments to assess registrants’ recent recovery experiences and SUD diagnosis in DSM-5, DD, and QOL. To be included in the current study, the registrants needed to complete an online assessment measuring their ED, sleep quality, and perceived stress, respond to one of the quarterly assessments within three months, and endorse two or more diagnostic criteria for DSM-5 SUD in lifetime history for at least one substance. A total of 139 IQRR registrants met the inclusion criteria. Of those registrants, 21 were excluded from the present analysis due to one or more of the following reasons: (a) unresolvable sleep data for scoring (i.e., supernumerary sleep durations; *n* = 14); and (b) missing and/or inconsistent demographic data across assessments (*n* = 8). Thus, the final sample comprised 118 participants.

A consent form describing the purpose of data collection was provided at the beginning of each assessment. Completing and submitting the assessment implied consent to research and publication. Each participant received 1500 points in the IQRR that could be redeemed for $15 compensation in the current study.

### Measures

#### Delay discounting

Individuals’ DD rates were measured using the 5-trial adjusting delay discounting task (ADDT) [65]. The participant was initially presented with a choice of receiving a hypothetical amount of $50 now or $100 in three weeks. The time delay for receiving $100 in the following trials were adjusted (ranging from 1 h to 25 years) according to their choices, while the monetary rewards remained unchanged to approach the point at which the two options are subjectively equal. Individual DD rate (*k*) estimates were obtained based on their responses at the fifth trial and were transformed using natural log to normalize the data and stabilize the variance.^1^

#### Effort discounting

Individuals’ ED rates were measured using a binary choice task adapted from ADDT, in which the time delays were replaced with corresponding amounts of typed words (i.e., 24 h = 24 words typed) [48]. Participants were first prompted to type an excerpt of 50 words for a word count reference and noted that one double-spaced page typically contains about 250 words, 10 pages contain 2,500 words, etc. Participants were then presented with a choice of earning a hypothetical amount of $50 for typing zero words or earning $100 for typing 504 words. The number of typed words in the following trials were adjusted (ranging from 1 to 219,150 words) according to their choices, while the monetary rewards remained unchanged to approach the point at which the two options are subjectively equal. Individual ED rate estimates were obtained based on their responses at the fifth trial and were transformed using natural log for the same reasons for DD.

#### World health organization quality of life (brief)

The brief version of the World Health Organization Quality of Life assessment measures an individual’s QOL in the physical health, psychological health, social relationships, and environmental domains [55]. This assessment contains 26 5-point Likert scale questions. The first two questions asked about one’s overall perceptions of QOL and personal health, while the remaining 24 inquired about QOL in the four domains. Each domain yields a raw score that was transformed to a 0–100 point interval, with a higher score indicating a greater QOL. This assessment has been evaluated collaboratively in 15 cultural settings over multiple years, showing strong internal consistency (Cronbach’s alpha for the domain scores ranged from 0.66 to 0.84) [55].

#### Pittsburgh sleep quality index

Individuals’ sleep quality over the previous month was measured using the Pittsburgh Sleep Quality Index (PSQI), a self-reported assessment composed of 19 free response and 5-point Likert scale questions [66]. There are 7 component scores produced: subjective sleep quality, sleep latency, sleep duration, habitual sleep efficiency, sleep disturbances, use of sleeping medication, and daytime dysfunction. The total score ranges from 0 to 21, and a reported score of five or greater indicated a significant sleep disturbance. The PSQI demonstrates strong internal consistency, with Cronbach’s alpha of 0.83 across its seven components [66].

#### Perceived stress scale

Individuals’ perceived stress over the previous month was measured using the Perceived Stress Scale (PSS), a self-reported assessment composed of 10 5-point Likert scale questions [67]. The total score ranges from 0 to 40, where 0–13 indicated low perceived stress, 14–26 indicated moderate perceived stress, and 27–40 indicated high perceived stress. A comprehensive review of 12 studies revealed that the PSS exhibits robust internal consistency, with Cronbach’s alpha ranging from 0.74 to 0.91 across the examined research [68].

#### Demographics

Participants were asked to self-report their age, sex, race, years of education, and household income. Participants who identified as Hawaiian (*n* = 1), Indian (*n* = 2), or Other (*n* = 1) were grouped together for statistical analysis. Household income were assessed using 21 intervals, ranging from less than $5,000 to $200,000 or more. The midpoint of each interval was used to estimate individual household income. For the highest interval without a specified range, $22,5000 was used as an estimate.

### Procedure

Data for the current study was collected using two surveys: (a) a quarterly IQRR survey in which participants answered a series of questions capturing recovery history, DSM-5 diagnostic criteria for SUDs, DD, QOL, and demographic information, presented in this order; and (b) a sleep/stress assessment, open from December 1, 2021, to January 12, 2022, in which participants completed the ED task, the PSQI, and the PSS, presented in this order. If a participant completed more than one quarterly assessment, the data most close to the date they provided their sleep quality and perceived stress information was used for the analysis. The assessments were administered using the Qualtrics survey platform.

### Data analysis

Participants’ characteristics, including demographics and behavioral measures, were described using mean, standard deviation, frequency, and percentages, where appropriate. Their lifetime SUDs, were grouped into Mono, Dual, and Poly based on the number of substances met the DSM-5 diagnostic criteria in lifetime history. Their remission status was defined as not meeting any SUD criteria other than craving in the past 3 months for all their lifetime SUDs [69]. Early and sustained remission were grouped together because only 3 participants in the sample were in early remission. The correlation between sleep quality and perceived stress was first conducted, followed by univariate linear regression analyses to determine the associations of sleep and stress with the measures of interest (i.e., DD, ED, and the four domains of QOL). An exhaustive model selection with multiple regression models was then performed for each measure of interest, including all variables (i.e., sleep quality and perceived stress) and covariates (i.e., demographics, lifetime SUDs, remission status, and years in recovery). The model with the lowest Bayesian Information Criterion (BIC) was considered optimal [70]. This analytical approach enabled us to identify the most parsimonious model and the key factors uniquely contributed to the total variance in the measure of interest. All coefficients in the optimal models were evaluated, noting that nonsignificant variables may also be retained.

Curtis et al. (2018) identified a positive association between sleep and DD without accounting for stress [39]. Given the similarity in self-report measures between our study and Curtis et al., an exploratory analysis was conducted to evaluate the potential impact of including stress as a covariate on this association. Specifically, two distinct regression models predicting DD with sleep were created, incorporating variables similar to those specified in Curtis et al. Stress was introduced as a covariate in one model while being omitted in the other.

The reported coefficients are unstandardized to preserve their original scale. All analyses were conducted using R version 4.0.3 [71].

## Results

Table 1 shows the descriptive analysis of the sample. The participants were 54% female, 77% white, and 19% black. On average, participants were 45.6 (*SD* = 16.8) years old, had 14.5 (*SD* = 4.2) years of education, reported an average of 59,419.5 (*SD* = 51,767.3) annual household income, and had been in recovery for an average of 14.3 (*SD* = 14.4) years. The average sleep quality score was 8.36 (*SD* = 4.37), which indicated a significant sleep disturbance was experienced among the participants (a reported score of five or greater indicates a significant sleep disturbance). The average perceived stress score was 17.70 (*SD* = 8.05), which fell within the moderate perceived stress range. Among the participants, 28.8%, 26.3%, and 44.9% met one, two, and three or more lifetime SUDs, respectively. The most prevalent SUDs in the sample were lifetime alcohol use disorder (79.7%) and cannabis use disorder (50.9%), followed by lifetime cocaine use disorder (33.9%), prescription pain relievers use disorder (33.1%), opioids use disorder (31.4%), stimulants use disorder (30.5%), hallucinogens use disorder (23.7%), tranquilizers/depressants use disorder (22.9%), inhalants use disorder (16.1%), and dissociative anesthetics use disorder (9.3%). At the time of assessment, 60.2% of the participants were in remission.

**Table 1:** Summary of sample characteristics

Characteristics (*N* = 118)	Mean (*SD*)/ frequency (%)	Median [Q1, Q2]
Age	45.6 (16.8)	45.5 [30.0, 60.0]
Female	64 (54%)	
Years of education	14.5 (4.2)	15.0 [12.0, 17.0]
Household income	59,419.5 (51,767.3)	45,000.0 [17500.0, 75,000.0]
Race		
Black	23 (19.5%)	
White	91 (77.1%)	
Other	4 (3.4%)	
Lifetime SUD		
Mono	34 (28.8%)	
Dual	31 (26.3%)	
Poly	53 (44.9%)	
Remission status		
Current SUD	47 (39.8%)	
Remission	71 (60.2%)	
Years in recovery	14.3 (14.4)	7.5 [3.0, 25.5]
Delay discounting	− 4.85 (3.32)	− 5.97 [− 7.14, − 3.23]
Effort discounting	− 4.58 (3.44)	− 5.00 [− 6.80, − 2.84]
WHOQOL-BREF		
Physical health	69.7 (15.9)	71.4 [60.0, 80.0]
Psychological health	66.3 (15.1)	66.7 [54.2, 76.7]
Social relationships	64.1 (18.4)	66.7 [53.3, 73.3]
Environment	73.8 (14.9)	76.3 [62.5, 85.0]
Sleep	8.36 (4.37)	7.00 [5.00, 12.00]
Stress	17.7 (8.05)	18.0 [13.3, 22.0]

The mean (standard deviation) and frequency (percentage) are reported where appropriate. Q1 = first interquartile. Q3 = third interquartile. *SUD* substance use disorder in DSM-5. *WHOQOL-BREF* World Health Organization Quality of Life Assessment (Brief)

The correlation between sleep quality and perceived stress was 0.51 (*p* < 0.001) in the current sample, confirming their interdependent relationship [24]. Tables 2 and 3 show the associations of sleep quality and perceived stress with the measures of interest in univariate linear regression analyses. As may be seen, the coefficients of sleep and stress were significant across all measures of interest except the coefficient of stress for ED.

**Table 2:** Univariate Linear Regression of Delay and Effort Discounting

	Delay discounting		Effort discounting	
	Coefficient	*p*	Coefficient	*p*
Sleep	0.28	< .001	0.16	0.030
Stress	0.14	< .001	0.07	0.068

**Table 3:** Univariate Linear Regression of Quality of Life

	Physical health		Psychological health		Social relationships		Environment	
	Coefficient	*p*	Coefficient	*p*	Coefficient	*p*	Coefficient	*p*
Sleep	− 2.35	< .001	− 1.46	< .001	− 1.23	.001	− 1.52	< .001
Stress	− 1.31	< .001	− 1.26	< .001	− 1.24	< .001	− 1.07	< .001

The results of model selections showed that sleep quality and/or perceived stress were explanatory variables for all measures of interest except ED when demographic and addiction-related variables were accounted for (Table 4). The optimal multiple linear regression models showed that lower sleep quality (i.e., higher sleep scores) was associated with higher DD rates (*t* = 2.11; *p* = 0.037; *f* = 0.20) and lower QOL in physical health (*t* = − 5.76; *p* < 0.001; *f* = 0.54). Higher perceived stress was associated with lower QOL in physical health (*t* = − 6.35; *p* < 0.001;* f* = 0.59), psychological health (*t* = − 7.21; *p* < 0.001; *f* = 0.67), social relationships (*t* = − 6.82; *p* < 0.001; *f* = 0.64), and environment (*t* = − 4.78; *p* < 0.001; *f* = 0.45).

**Table 4:** Multiple Linear Regression of Variables Related to the Measures of Interest

Measures of interest	Variables	Coefficient	Std. Error	*t*	*p*	Partial Cohen’s *f* (Adj. *R*^2^)
Delay discounting	(Intercept)	2.12	2.47	0.86	.392	(.33)
	Sleep	0.13	0.06	2.11	.037	.20
	log_10_(income)	− 1.21	0.50	− 2.40	.018	.23
	log_10_(years in recovery + 1)	− 1.59	0.63	-2.52	.013	.24
	Remission status	− 1.75	0.61	− 2.88	.005	.27
Effort discounting	(Intercept)	− 2.77	0.46	− 6.10	< .001	(.18)
	Remission status	− 3.00	0.59	− 5.12	< .001	.48
QOL—physical health	(Intercept)	98.20	2.54	38.72	< .001	(.58)
	Sleep	− 1.50	0.26	− 5.76	< .001	.54
	Stress	− 0.90	0.14	− 6.35	< .001	.59
QOL—psychological health	(Intercept)	79.69	3.57	22.32	< .001	(.50)
	Stress	− 1.02	0.14	− 7.21	< .001	.67
	Remission status	7.87	2.32	3.39	< .001	.32
QOL—social relationships	(Intercept)	42.12	12.76	3.30	.001	(.37)
	Stress	− 1.16	0.17	− 6.82	< .001	.64
	Sex—female	6.05	2.71	2.24	.027	.21
	log_10_(income)	8.64	2.61	3.31	.001	.31
QOL—environment	(Intercept)	37.00	9.67	3.83	< .001	(.50)
	Stress	− 0.68	0.14	− 4.78	< .001	.45
	Years of education	0.72	0.24	3.03	.003	.28
	log_10_(income)	7.33	1.92	3.83	< .001	.36
	Remission status	8.41	2.31	3.65	< .001	.34

The variables associated with each measure of interest were determined through an exhaustive model selection. The reference group for Sex was those who were male. *QOL* quality of life

Worth noting, the results of our analyses showed that female had higher QOL in the social relationships domain than male. Higher household income was associated with lower DD rates and higher QOL in the social relationships and environment domains. Furthermore, higher education levels were associated with higher QOL in the environment domain. Remission status was associated with lower DD and ED rates and higher QOL in the psychological and environment domains. Longer years in recovery were associated with lower DD rates.

Finally, to assess whether different covariates would influence the findings in Curtis et al., [39], we conducted an exploratory analysis using variables similar to those outlined in their study for comparison (see Table 5) [39]. Intriguingly, the model that did not account for stress showed a significant association between sleep and DD (*t* = 2.56; *p* = 0.012; *f* = 0.24), indicating robust results. However, upon incorporating stress as a covariate, this association became nonsignificant (*t* = 1.60; *p* = 0.112; *f* = 0.15). Worth noting, adding stress did not improve the overall model fit, as the coefficient for stress was nonsignificant (*t* = 1.60; *p* = 0.112; *f* = 0.15). From a parsimonious perspective, the finding in our exploratory analysis aligns with the exhaustive model selection, where a significant association between sleep and DD was observed*.*

**Table 5:** Predictors of Delay Discounting with and without Accounting for Stress

Variables	Coefficient	Std. error	*t*	*p*	Partial Cohen’s *f* (Adj. *R*^2^)
(Intercept)	4.16	2.73	1.52	.130	(.28)
log_10_(income)	− 1.46	0.52	− 2.80	.006	.26
Education	− 0.04	0.06	− 0.57	.569	.05
Age	− 0.06	0.02	− 3.90	< .001	.37
Sex—female	− 0.67	0.53	− 1.28	.204	.12
Sleep	0.17	0.07	2.56	.012	.24
(Intercept)	3.18	2.78	1.14	.255	(.29)
log_10_(income)	− 1.43	0.52	− 2.77	.007	.26
Education	− 0.04	0.06	− 0.57	.571	.05
Age	− 0.06	0.02	− 3.57	< .001	.34
Sex—female	− 0.70	0.52	− 1.33	.187	.13
Sleep	0.12	0.07	1.60	.112	.15
Stress	0.06	0.04	1.60	.112	.15

The variables were selected to resemble the model reported in Curtis et al., [39]. The reference group for Sex was those who were male

## Discussion

This study examined the associations of sleep quality and perceived stress with DD, ED, and QOL in individuals recovering from SUDs. Consistent with the hypotheses, in univariate linear regression analyses, poorer sleep quality and higher perceived stress were associated with higher DD rates, higher ED rates, and lower QOL, although the association between stress and ED was nonsignificant. Despite showing a significant association with DD, stress failed to emerge as an explanatory variable in the optimal multiple linear model when other variables were considered. Similarly, when demographic and addiction-related variables were considered, neither sleep nor stress emerged as an explanatory variable of ED. Regarding QOL, both sleep and stress were explanatory variables of physical health in the optimal model. However, in the psychological health, social relationship, and environment domains, only stress emerged as an explanatory variable.

Interestingly, although we observed that both poorer sleep quality and high perceived stress were significantly associated with higher DD among individuals recovering from SUDs, sleep quality rather than perceived stress emerged as an explanatory variable of DD. The association between stress and DD is well-established in the addiction recovery population [41–45]. Our findings indicate sleep explained a greater variance of DD than stress in the current sample and highlights the importance of considering sleep while investigating the association between stress and DD. Due to the interdependent status of sleep and stress, this implication may apply to other addiction recovery research, in which stress has been demonstrated to correlate with a diverse pool of measures, including overall childhood maltreatment [72], depressive symptoms [73], social exclusion factors [74], coping responses [75], contentment [76], and spirituality [75, 76].

Our findings also contribute to the existing literature, which has yielded mixed results regarding the association between sleep and DD. Both our study and Curtis et al., [39] utilized self-report measures, and we replicated their finding of a positive association between sleep and DD in the exploratory analysis. However, this association became nonsignificant when stress was included in the regression model as a covariate, despite stress not significantly explaining the variance in DD beyond what is already accounted for in the model. These results highlight a main challenge when using stepwise regression to tease out the unique contributions of sleep and stress. Specifically, the order of introducing the variables of interest in the model is arbitrary and might lead to different interpretations of the data, even though this decision is typically based on theory. In this case, if stress is retained while sleep is omitted in the first model, one may conclude that stress is positively associated with DD, and sleep does not explain variances beyond what has been included. In contrast, the exhaustive model selection method aims to identify the key factors uniquely contributing to the total variance in the measure of interest and does not restrict which variable would emerge in the optimal model. The selected model will only retain stress and sleep if their contributions go beyond the other variables included in the search space. Thus, when simultaneously examining sleep and stress to distinguish their unique impacts on key variables, this data-driven analytical approach (i.e., exhaustive model selection) may be preferable over stepwise regression. Beyond these considerations, two noteworthy distinctions between the present study and prior research should be acknowledged, as they might contribute to the variations in findings. Firstly, unlike previous studies that predominantly involved healthy adults, our investigation focused on individuals recovering from SUDs. This distinction suggests that the observed association may be specific to this population. Secondly, our study relied on self-report measures, unlike many studies with null findings, which typically involve experimental manipulations assessing change in DD after sleep deprivation.^2^ The association between sleep and DD may indeed exist, with DD affecting sleep [77]. Further research is warranted to confirm these relationships.

In contrast to DD, the associations of sleep quality and perceived stress with ED were marginal in the present study. This observation contrasts with findings demonstrating that poorer sleep quality and reduced sleep duration are associated with less preference for high effort and high rewards, but not DD among healthy young adults [36, 49]. Further, while the literature on the impact of stress on ED in humans is scant, in rats, stress exposure is related to decreases in preference for high effort, high-rewards [51]. Although remission status explained a greater variance of ED than sleep and stress in our sample, additional research is necessary to establish the relationships between sleep, stress, and ED in SUD recovery.

One key finding of the present study is that perceived stress is a unique explanatory variable of QOL and is negatively associated with all domains among individuals in recovery from SUDs, which is consistent with prior research on different populations. For example, a systematic review by Ribeiro et al., [61] reported a negative relationship between stress and QOL among university students through the decline of different physical and mental health aspects [61]. Pokhrel et al., [78] reported that high-stress levels were associated with lower scores across all QOL domains, including the physical and psychological domains, among HIV-positive individuals [78]. Ames et al., [57] reported that stress was a significant predictor of QOL in the physical and mental health domains among low-income patients with established hypertension, even after statistically controlling for age and the number of chronic illnesses [57]. Moreover, previous research reported that perceived stress mediates the association between coping strategies and QOL [79] and is associated with symptoms of psychiatric disorders, such as anxiety and depression [80, 81] and higher levels of nicotine dependence [82]. Future longitudinal research investigating the association between changes in stress and QOL over time in SUD recovery populations is warranted.

Our data also shows that sleep quality is a unique explanatory variable of QOL in the physical health domain among individuals in recovery from SUDs. This finding corroborates previous research reporting that the co-occurrence of sleep problems and substance misuse is common and may result in problems with physical activities and health problems (e.g., pain) [60]. Sleep problems can negatively affect health-related QOL among individuals with SUDs [63], and those who experience sleep disturbances are at higher risk of chronic diseases such as obesity, diabetes, and cardiovascular diseases [83]. Noticeably, sleep failed to emerge as an explanatory variable of QOL in the psychological health, social relationships, and environment domains. Our results showed stress may be a confounder that explains the significant associations between sleep and QOL observed in the prior investigations [59, 62–64, 83, 84]. These relationships may also depend on the population studied. Lee et al., [60] reported a significant association between sleep quality and QOL after controlling for individual stress levels and demographics in healthy adults [60]. Additional research is needed to improve our understanding of sleep, stress, and QOL relationships.

The findings of this study have important implications for SUD treatment. First, the study confirms the relationships between sleep quality, perceived stress, and other known factors in addiction recovery. This suggests that SUD treatment should address sleep and stress in addition to substance use itself, consistent with the recommended practices [6, 7]. Second, the study found that sleep quality and perceived stress are associated with different aspects of SUD recovery. Specifically, poorer sleep quality and higher perceived stress were associated with higher DD rates and lower QOL, particularly in the physical health domain. These findings suggest that sleep and stress interventions may be particularly beneficial for reducing DD rates and improving physical health in people recovering from SUDs. Third, the study found that stress emerged as a more consistent explanatory variable of QOL in SUD recovery than sleep quality after controlling for other factors. This suggests that stress management interventions may be particularly important for SUD treatment and service. However, recognizing sleep quality and stress are interdependent, addressing both factors may be most effective. By integrating sleep and stress interventions into SUD treatment and service, providers can help individuals recover from SUDs and promote healthier, more fulfilling lives.

Although the findings have significant clinical and research implications, the study lacks data on psychiatric disorders. This absence may have impacted the observed relationships among individuals recovering from SUD. Sleep disturbance is a primary symptom among multiple psychiatric disorders such as depression and post-traumatic stress disorder. Similarly, stress responses vary across psychiatric disorders [85]. The extent to which psychiatric disorders confounded the observed relationships is unclear. To complicate this picture even further, the comorbidity between substance use and psychiatric diagnoses may also change the observed relationships. For example, previous research has suggested that individuals with comorbid major depressive disorder and SUD have more sleep problems and poorer QOL than those without comorbidity [86]. Given that about half of those who experience an SUD will also experience mental illnesses during their lives [87], future research should take into account both psychiatric disorders and their comorbidity with SUDs.

Additional limitations of the study should be acknowledged. Firstly, the study used cross-sectional data, meaning that causal relationships between the variables cannot be established. Nevertheless, this study identified important variables that should be considered in future longitudinal investigations of addiction recovery. Secondly, our findings relied on a convenience sample, which mainly consisted of white, middle-aged, and educated individuals, possibly introducing self-selection bias. The representativeness of the sample is further compromised by its size in relation to the heterogeneity within SUD populations. Although these concerns are somewhat mitigated by observing multiple established relationships such as the correlation between sleep and stress and a significant association between stress and DD, additional research is necessary to validate the robustness of our findings. Finally, the relationships among the variables may be non-linear while linear regression models were utilized for the analysis. For example, sleep quality may influence an individual’s QOL only when it reaches a certain threshold. This concern, however, was ameliorated by noting that non-linear relationships were not apparent in the visual plots. In light of the mentioned limitations, this exploratory study underscores the importance of considering both sleep and stress factors in addiction recovery. Moreover, incorporating these findings into future research endeavors on recovery may aid in pinpointing the underlying mechanisms, thereby contributing to the development of more comprehensive and effective interventions in the field.

## Data Availability

All data, analysis code, and research materials are available by emailing the corresponding author. This study’s design and its analysis were not pre-registered.

## Footnotes

1 The DD rate was estimated with a hyperbolic-decay model (Mazur, 1987): *V* = *A* / (1 + *kD*), where *V* is the present, discounted value, *A* is the amount of the delayed reward, *D* is the delay to its receipt, and *k* is a parameter governing the rate at which value is discounted with delay [88]

2 Law and Rasmussen (2023) is the only exception. Their study also utilized self-report measures and addressed both sleep and stress. However, in addition to finding no association between sleep and monetary DD in their sample, the correlation between stress and DD was also nonsignificant, contradicting the established literature [40].
